# Novel Compound Heterozygous *TMPRSS15* Gene Variants Cause Enterokinase Deficiency

**DOI:** 10.3389/fgene.2020.538778

**Published:** 2020-09-11

**Authors:** Lan Wang, Dan Zhang, Cheng Fan, Xiaoying Zhou, Zhifeng Liu, Bixia Zheng, Li Zhu, Yu Jin

**Affiliations:** ^1^Department of Gastroenterology, Children’s Hospital of Nanjing Medical University, Nanjing, China; ^2^Digestive Department, Children’s Hospital of Guiyang, Guiyang, China; ^3^Nanjing Key Laboratory of Pediatrics, Children’s Hospital of Nanjing Medical University, Nanjing, China

**Keywords:** *TMPRSS15*, enterokinase deficiency, chronic diarrhea, missense variant, splicing

## Abstract

**Background:**

Enterokinase deficiency (EKD) is a rare autosomal recessively inherited disorder mainly characterized by diarrhea, hypoproteinemia and failure to thrive in infancy. Loss-of-function variants in the *TMPRSS15* gene cause EKD.

**Methods:**

We report the clinical manifestations and molecular basis of EKD in a Chinese child. We investigated *in vitro* two *TMPRSS15* variants: the c.1921G > A as a possible splicing variant by minigene assay; the c.2396T > A(p.Val799Asp) as a missense change by protein expression analysis, enterokinase activity and effect on cellular localization.

**Results:**

The proband presented with intractable diarrhea accompanied by vomiting, failure to thrive and hypoproteinemia in his second year. Genetic analysis showed that the patient was compound heterozygous for two variants in the *TMPRSS15* gene: c.[1921G > A];[2396T > A]. The c.1921 G > A variant may change the glutamic acid 641 into lysine; this change is predicted to be benign by bioinformatics analysis. However, it was predicted to disrupt the splicing donor site. Our minigene assay revealed that c.1921G > A caused the skipping of exon 16. The c.2396T > A(p.Val799Asp) change in the serine protease domain predicted to be deleterious hitting an evolutionary conserved amino acid. Functional studies *in vitro* revealed that the p.Val799Asp variant decreased the total expression level of *TMPRSS15* by 29%, and the enterokinase activity of p.Val799Asp mutants was decreased by 37%, compared with that of wild type.

**Conclusion:**

We reported an EKD patient with novel compound heterozygous variants in the *TMPRSS15* gene, expanding the genotypic and phenotypic spectrum of EKD. The functional characterization *in vitro* demonstrated that the c.1921G > A variant alters pre-mRNA splicing and the p.Val799Asp variant leads to a decrease in protein expression and enzyme activity.

## Introduction

Enterokinase deficiency (EKD, OMIM#226200), also named congenital enteropeptidase deficiency (CEP), is an extremely rare autosomal recessive genetic disease mainly characterized by severe chronic diarrhea after birth, hypoproteinemia, and failure to thrive. Loss-of-function variants in the transmembrane protease serine 15 (*TMPRSS15*) gene (OMIM#606635) are responsible for EKD. The lack of enterokinase (EK) prevents the activation of trypsinogen, which leads to a disorder of intestinal protein absorption (Holzinger et al., 2002). EKD was first described by Hadorn et al. (1969), and only approximately 10 cases have been reported worldwide thus far. To date, according to the Human Genome Mutation Database (HGMD), only four variants have been described in the *TMPRSS15* gene. Here, we reported a case of EKD caused by novel compound heterozygous variants in the *TMPRSS15* gene: c.[1921G > A];[2396T > A]. The effect of the c.1921G > A variant on splicing was investigated by a minigene (pSPL3 splicing) assay *in vitro*. We also characterized the protein expression, enzymatic activity and cellular localization of the c.2396T > A (p.Val799Asp) variant *in vitro*.

## Materials and Methods

### Patient

The proband (II-2) was a Miao Chinese boy who was admitted to the hospital for diarrhea for more than 1 month in his second year. The diarrhea was described as yellow watery stool with a little mucus, and it occurred more than 10 times a day and was accompanied by vomiting between 2 and 3 times a day. Physical examination demonstrated the following: body weight, 8500 g; apparent malnutrition; subcutaneous fat, 0.4 cm; poor skin elasticity; poor spirit; and apparent mild dehydration. The proband’s birth weight (3.5 kg) and birth history were normal. He received surgical treatment for anal stenosis in infancy. He was breastfed until being switched to formula feeding at the age of 1 and received complementary foods normally. His brother was 7 years old when he became sick. There was no similar family history. Laboratory investigations were performed to evaluate the proband’s chronic diarrhea and failure to thrive and found the following: the serum albumin level decreased (32.8 g/L); the white blood cell count was 14.47 × 10^9^/L; the red blood count was 6.46 × 10^12^/L; the C-reactive protein (CRP), alanine aminotransferase (ALT), aspartate aminotransferase (AST), and bilirubin levels in serum were normal (CRP: < 4 mg/L; ALT: 9 U/L; AST: 39 U/L; TBIL: 6.33 μmol/L; DBIL: 5.6 μmol/L; IBIL: 0.7 μmol/L); the K^+^ concentration was 3.0 mmol/L, the Na^+^ concentration was 125 mmol/L, and the Cl^–^ concentration was normal; stool analysis revealed a few fat globules and no parasites; and stool cultures were negative for enteropathogens. No obvious abnormality was found in abdominal computed tomography or pathological biopsy results. He was treated with probiotics and montmorillonite powder, provided intravenous nutrition and subjected to correction of electrolyte disorder throughout the course. After admission, he was subjected to fasting, during which he vomited less, but the diarrhea continued. The stool was tested again, and increased white and red blood cell counts were detected. Therefore, we believed that the child had an intestinal infection, and he was treated with antibiotics. He was fed extensively hydrolyzed formula (eHF) discontinuously. However, his symptoms of diarrhea were not completely alleviated. On the 10th day of hospitalization, the child developed a pulmonary infection. The parents suspended treatment on the 16th day of the hospitalization and the patient was discharged to home care. The child passed away from the illness 2 weeks after discharge. The timeline of clinical phenotype development is shown in Supplementary Figure S1.

### Genetic Analysis

Peripheral blood samples were collected from the patient and his family members and sent to MyGenostics (Beijing, China) for whole exome sequencing (WES). Firstly, Genomic DNA was isolated using a DNA isolation kit (Tiangen, China) and sheared into fragments and then hybridized with the xGen Exome Research Panel v1.0 probe sequence capture array from IDT (Integrated Device Technology, United States). Sequencing reads were mapped to the GRCh37/Hg19 reference genome via Burrows-Wheeler Aligner (BWA) software. All identified variants were annotated using the 1000 Genomes Project (Chinese), dbSNP, and Genome Aggregation Database (gnomAD). The candidate variants were classified according to the ACMG (American College of Medical Genetics and Genomics) criteria and confirmed by direct Sanger sequencing. Written informed consent of the study was obtained from the parents. The study protocol was approved by the ethics committee of the Children’s Hospital of Nanjing Medical University. The *TMPRSS15* reference sequence is NM_002772.2.

### Bioinformatics Predictions

We used the following tools to predict whether an amino acid substitution affected the function of EK: Polymorphism Phenotyping v2 (PolyPhen-2)^1^, Sorting Intolerant From Tolerant (SIFT)^2^ and Functional Analysis Through Hidden Markov Models (FATHMM)^3^. The ConSurf Server^4^ was used to detect the conservatism of variants. To analyze the potential effect of variants on splicing, we used Human Splicing Finder (HSF)^5^, NetGene2^6^ and NNSplice^7^.

### Construction of the Minigene_*TMPRSS15*_ex Plasmids

We extracted genomic DNA from peripheral leukocytes of the patient who carried a heterozygous variant of c.1921G > A using a DNA isolation kit (Tiangen, China). We used pSPL3 vector, a generous gift from Dr. Irene Bottillo (University of Sapienza, Italy) and Dr. Leping Shao (University of Qingdao, China). Wild-type and mutant genome fragments of *TMPRSS15* including exon 16 (141 bp), intron 15 (121 bp) and intron 16 (173 bp) could be amplified using the patient DNA directly by Phanta Super-Fidelity DNA Polymerase (Vazyme, China). The primers used in this step were edited by Primer 5 software^8^ and were incorporated restriction endonuclease sites to the 5 ends (Forward 5′-accagaattctggagctcgagCTATGAGCGAGCCATGTAAC-3′; reverse 5′-atcaccagatatctgggatccCAGGTAACGGTACTACAGAT-3′). All fragments were cloned into the pSPL3 vector with the *Xho*I and *Bam*HI using ClonExpressTM II One Step Cloning Kit (Vazyme, China). To select Minigene_*TMPRSS15*_ex16-WT and Minigene_*TMPRSS15*_ex16-MUT (c.1921G > A), constructs were sequenced by Sanger sequencing.

### Construction of Overexpression Plasmids Encoding Enterokinase

The wild-type human *TMPRSS15* cDNA (GenBank NM_002772) and human *PRSS3* cDNA (GenBank NM_007343) were purchased (YouBio, China) and were cloned into pcDNA3.1-3xFlag and pcDNA3.1-HA vectors respectively using Clon Express Entry One Step Cloning Kit (Vazyme, China). Wild-type plasmid pcDNA3.1-3xFlag-*TMPRSS15* and pcDNA3.1-HA-*PRSS3* were successfully constructed. To obtain the vector of pcDNA3.1-3xFlag-*TMPRSS15*-MUT (c.2396T > A), we performed site-directed mutagenesis with the PCR-based *Dpn*I-treatment method (Vazyme, China). The primers are as follows: c.2396T > A-forward 5′-TGGGaTGTGGGTCTGTATTATGGCGGCCGACT-3′ and c.2396T > A-reverse 5′-TACAGACCCACAtCCCAGGGCC AGGCCCCTTC-3′. The mutagenesis was confirmed by bidirectional sequencing.

### Cell Culture and Transfection

HEK293 (human embryonic kidney 293), A549 (human lung epithelium carcinoma cell line), or HeLa (human cervical carcinoma) cells were seeded in 6-well plates with 2 mL of Dulbecco’s Modified Eagle’s Medium (DMEM) in each well at 37°C in an atmosphere of 5% CO_2_. After the cells were 50–70% confluent, cells were transfected with purified plasmids using Lip2000 (Invitrogen) in accordance with the manufacturer’s protocols.

### *In vitro* Minigene Splice Assay

HEK293, A549 or HeLa cells were transfected with purified plasmids containing Minigene_*TMPRSS15*_ex16-WT and Minigene_*TMPRSS15*_ex16-MUT. 24 h after transient transfection, total RNA was extracted from cells using Trizol Reagent (Takara, Japan). First cDNA strand was reversely transcribed using the SuperScript III transcriptase (Invitrogen) together with random hexanucleotide primers. Two microliters of each cDNA were amplified by TaKaRa Ex Taq (Takara, Japan) with primers located in the two cassette exons of pSPL3 vector. The primers sequences are as follows: Forward SD 5′-TCTGAGTCACCTGGACAACC-3′ and reverse SA 5′-ATCTCAGTGGTATTTGTGAGC-3′. PCR products were analyzed by agarose gel electrophoresis and proven by sequencing of extracted DNA. Gel was containing 1.5% agarose in Tris-acetate-EDTA buffer.

### Real-Time Quantitative Polymerase Chain Reaction (RT-qPCR)

HEK293 cells were transfected with pcDNA3.1-3xFlag-*TMPRSS15* or pcDNA3.1-3xFlag-*TMPRSS15*-MUT. Total RNA was extracted from cells using Trizol Reagent (Takara, Japan) and cDNA was reversely transcribed using the SuperScript III transcriptase (Invitrogen). RT-qPCR was performed on a Quant Studio 3 real-time PCR machine (Applied Biosystems, United States) using the SYBR Green master mix (Vazyme, China). Fold changes were calculated using the method of 2^–Δ^ ^Δ^ ^*Ct*^, and expression levels normalized to the average of the housekeeping genes glyceraldehyde-3-phosphate dehydrogenase (*GAPDH*). The primers sequences are shown as follows: *TMPRSS15*-forward 5′-CAACATTCAGTTCTACGAAC-3′, *TMPRSS15*-reverse 5′- TGCCATATGCTCTGGATTCT-3′, *GAPDH*-forward 5′-AGGTCGGTGTGAACGGATTTG-3′ and *GAPDH*-reverse 5′-GGGGTCGTTGATGGCAACA-3′.

### Western Blot Analysis

HEK293 cells were transfected with purified plasmids containing either pcDNA3.1-3xFlag-*TMPRSS15* or pcDNA3.1-3xFlag-*TMPRSS15*-MUT. Total proteins were collected after 48 h by RIPA buffer (Beyotime, China) which contained 1% proteasome inhibitor (Sigma, United States). Protein concentration was determined using a Micro BCA protein assay kit with bovine serum albumin as a standard (Thermo Fisher Scientific, United States). Then the proteins were separated by 8% SDS-PAGE and transferred onto PVDF membrane. The membranes were blocked by TBS-T (0.1% Tween 20 in TBS) containing 5% non-fat milk for 1 h at room temperature, and then incubated with primary antibodies against Flag (1: 3000, Sigma, United States), GAPDH (1: 3000, ProTech, China) overnight at 4°C, followed by the incubation of HRP-labeled secondary antibodies at room temperature for 1 h. Membranes were visualized by chemiluminescence reaction. Band intensity was quantified using Image J software (NIH, Bethesda, MD, United States).

### Immunofluorescence

EK is a transmembrane protein. To determine the localization of EK^*Val*799*Asp*^, HEK293 cells were grown in 12-well culture plates containing glass slides, cultured and transfected with pcDNA3.1-3xFlag-*TMPRSS15* or pcDNA3.1-3xFlag-*TMPRSS15*-MUT according to the above method. After 48 h, the cells were fixed with acetone for 20 min at 4°C. The cells were sealed 5% BSA (prepared by 1 × PBS) at 37°C for 1 h. Mouse anti-Flag (1:400, Sigma, United States) and rabbit anti-Na^+^/K^+^-ATPase antibodies (1:400, Abcam, United States) were incubated overnight at 4°C. The secondary antibodies were added and incubated for 2 h at 37°C. Imaging was performed on an inverted confocal laser scanning microscope. The excitation wavelength was 488 nm for FITC and 543 nm for Cy3.

### Trypsin Activity Assay

Trypsin activity was monitored as the amount of pnitroaniline (pNA) released from a specific substrate according to the manufacturer’s protocol [Trypsin Activity Assay kit (Abcam, United States)], which was an established method in the literature (Hayashi et al., 2018). HEK293 cells were transfected with pcDNA3.1-3xFlag-*TMPRSS15*, pcDNA3.1-3xFlag-*TMPRSS15*-MUT or pcDNA3.1-HA-*PRSS3*. Cells were harvested in PBS and then lysed and homogenized by sonication to collect total proteins. Proteins were quantified according to the method mentioned above. Based on western blot, the protein mass of wild-type and mutants was quantified equally (EK^*WT*^: EK^*Val*799*Asp*^, 7: 10). The sufficient HEK293 cell lysates exogenously expressing trypsinogen (*PRSS3*) were incubated with the lysates of EK^*WT*^, EK^*Val*799*Asp*^ or vector respectively. Samples were measured at 405 nm and at 25°C.

## Results

### Genetic Findings

**FIGURE 1:**
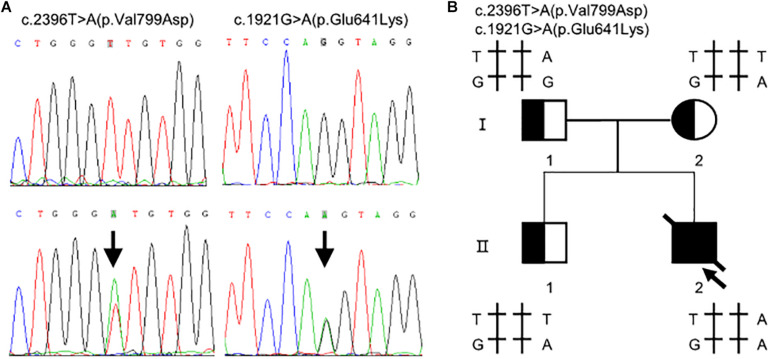
Compound heterozygous variants in the *TMPRSS15* gene in a patient with enterokinase deficiency. **(A)** The variant c.1921G > A is located in exon 16 and the missense variant c.2396T > A represents a p.Val799Asp change in exon 21. The arrows indicate the heterozygous base. **(B)** Family pedigree: The arrow indicates the proband. The slash indicates that the individual was dead. The squares represent the proband, his older brother and his father, and the circle represents the mother.

Whole exome sequencing identified two heterozygous variants in *TMPRSS15* gene: c.[1921G > A];[2396T > A] (Figure 1A). Subsequently, Sanger sequencing was performed on family members (Figure 1B). His mother (I-2) and older brother (II-1) carried the variant c.1921G > A. His father (I-1) carried the heterozygous variant c.2396T > A. The two variants have not been reported in the literature or HGMD and are rare in the GnomAD (ver.2.1.1) database (c.1921G > A, rs2273204, allelic frequency 355/282370, 3 homozygotes; c.2396T > A, rs764937713, allelic frequency 2/251488).

### Bioinformatics Prediction

**FIGURE 2:**
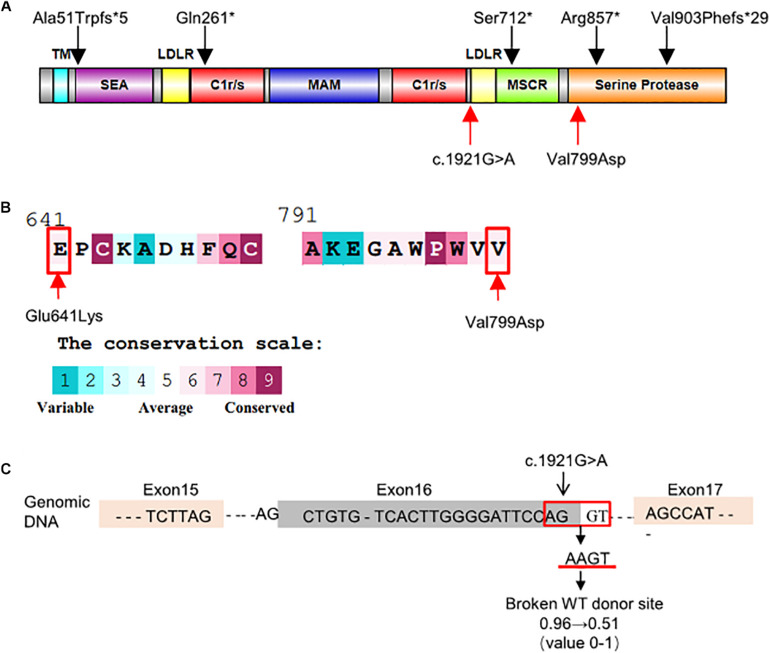
Schematic representation and bioinformatics predictions of *TMPRSS15* variants. **(A)** Schematic representation of the human EK protein and domain composition. The black arrows represent the variants reported in HGMD and ClinVar. The red arrows indicate the locations of the variants identified in this study. TM, transmembrane domain; SEA, domain found in sea urchin sperm protein, enterokinase, agrin; LDLR, LDL receptor–like domain; C1r/s, complement component C1r-like domain; MAM, meprin-like domain; MSCR, macrophage scavenger receptor-like domain (Zheng et al., 2009). **(B)** Evolutionary conservation of amino acids in EK determined by ConSurf Server. The grades varying from 1 to 9 indicate the extent of conservation of the amino acid throughout evolution, with grade 1 being the least and grade 9 being most conserved. p.Glu641Lys and p.Val799Asp were located in conserved regions with a score of 6. **(C)** HSF and NetGene2 predicted that the c.1921G > A was highly likely to destroy the splicing donor. NNSplice showed the variant decreased the donor score from 0.96 to 0.51.

The c.1921G > A variant, located in exon 16, was predicted as a missense variant resulting in the change of glutamic acid (E) to lysine (K) at codon 641 of EK (p.Glu641Lys). This amino acid change was predicted benign by PolyPhen-2 (0.063) and SIFT (0.42). However, HSF, NetGene2, and NNSplice predicted that it was a splicing variant having a high probability of destroying the splicing donor site (Figure 2C). Thus, exon 16 skipping may lead to a protein lacking 47 amino acids in the LDL receptor-like (LDLR) domain (Figure 2A).

The *TMPRSS15* variant c.2396T > A, located in exon 21, caused a valine (V)-to-aspartic acid (D) change at amino acid 799 of EK (p.Val799Asp). This variant is located in the serine protease domain and may affect EK activity (Figure 2A). *In silico* analysis predicted that this variant was likely harmful to protein function. The SIFT, PolyPhen_2 and FATHMM scores were 0.16 (harmful), 0.996 (harmful), and 2.46 (damaging), respectively. In addition, the result of the ConSurf analysis showed the p.Val799Asp variant is evolutionary conserved (Landau et al., 2005; Figure 2B).

### *In vitro* Minigene Splice Assay

**FIGURE 3:**
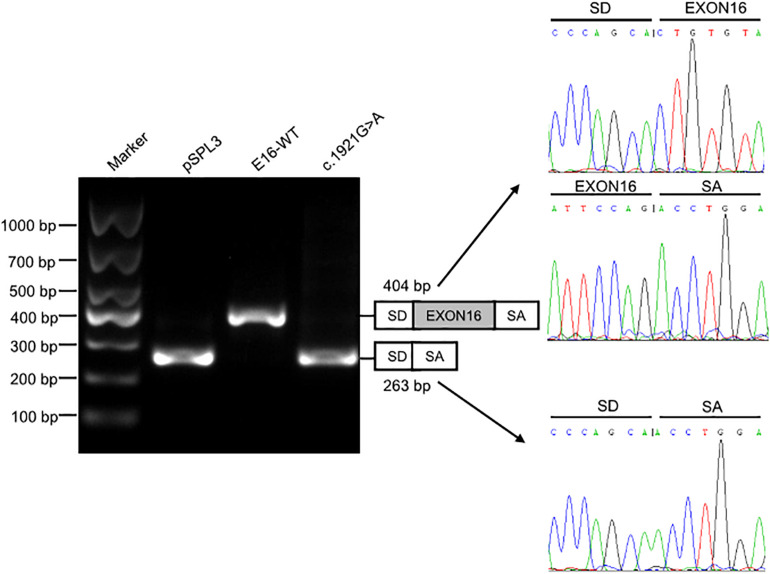
Splicing assay of the c.1921G > A variant in *TMPRSS15* gene by a minigene assay. Gel electrophoresis of the RT-PCR product of minigene transcripts in HEK293 cells. Lane 1: DL1000 Plus DNA Marker (Vazyme, China); lane 2: pSPL3 (263 bp); lane 3: E16-WT (404 bp); lane 4: c.1921G > A (263 bp). The two fragments were directly sequenced (right panel).

We investigated the effect of c.1921G > A on splicing using an *in vitro* minigene splicing assay, based on the pSPL3 exon trapping vector. The pSPL3 vector containing a conventional expression system with two cassette exons has functional splice donor and acceptor sites. To create minigene expression constructs (Minigene_*TMPRSS15*_ex16-WT and Minigene_*TMPRSS15*_ex16-MUT), genomic fragments of interest were inserted into the pSPL3 vector. After the minigene constructs were transfected into HEK293 cells, total RNA was extracted and transcribed to cDNA. PCR was performed using vector exon-specific primers and then visualized on an agarose gel. As a result, compared with the wild-type plasmids (estimated 404 bp), Minigene_*TMPRSS15*_ex16-MUT yielded only one smaller RT-PCR product (estimated 263 bp) corresponding to exon exclusion, which was determined by Sanger sequencing (Figure 3). The same results were obtained in A549 and HeLa cells (Supplementary Figure S2A). Taken together, these results suggested that the c.1921G > A is a splicing variant causing exon 16 skipping.

### Protein Expression and Enterokinase Activity of the Wild Type and p.Val799Asp Variant *in vitro*

**FIGURE 4:**
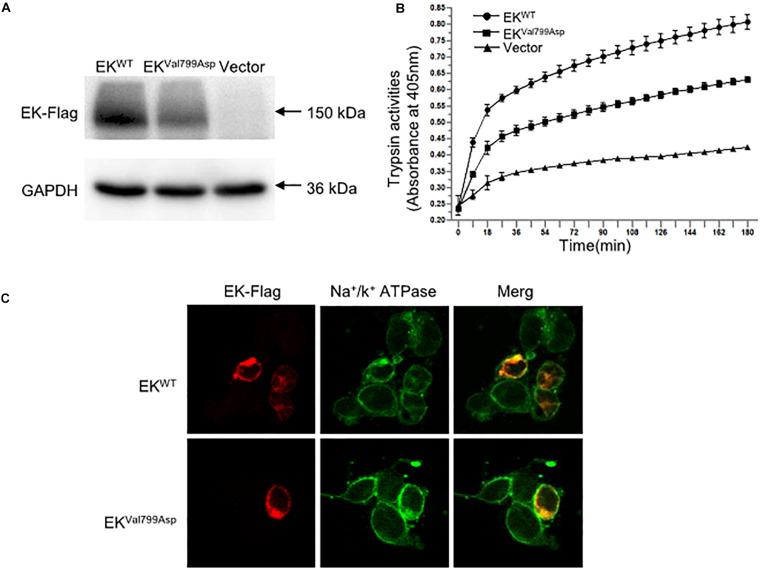
The expression, enterokinase activity and cellular localization of EK^*WT*^ and EK^*Val*799*Asp*^ *in vitro*. **(A)** The protein expression of EK-Flag was determined by western blotting. **(B)** Lysates of HEK293 cells exogenously expressing trypsinogen (*PRSS3*) were incubated with lysate of EK^*WT*^, EK^*Val*799*Asp*^ or vector, respectively. The amount of pNA released from the specific substrate in the mixture was measured at 405 nm as a function of trypsinogen activation (trypsin activity). **(C)** Cellular localization of EK-Flag in HEK293 cells was determined by immunofluorescence. All experiments were conducted in triplicate in three independent experiments.

To further verify the pathogenicity of the p.Val799Asp missense variant, pcDNA3.1-3xFlag-*TMPRSS15* or pcDNA3.1-3xFlag-*TMPRSS15*-MUT was transiently expressed in HEK293 cells. First, we examined the expression level of EK in HEK293 cells transfected with wild-type and mutant constructs by western blotting. In transiently transfected HEK293 cells, the RT-qPCR results showed that the transfection efficiency of the wild-type and mutant constructs was at the same level (Supplementary Figure S2B). The Flag-specific monoclonal antibody recognized a protein band (150 kDa) in cells transfected with wild-type and mutant constructs, and the mutant constructs caused a 29% decrease in total EK expression (Figure 4A).

We examined the subcellular distribution of EK^*WT*^ and EK^*Val*799*Asp*^ in HEK293 cells by immunofluorescence. As shown in Figure 4C, EK^*WT*^ and EK^*Val*799*Asp*^ both predominantly colocalized with the Na^+^/K^+^-ATPase (a plasma membrane marker), indicating a cellular membrane location. In addition, we incubated HEK293 cell lysates expressing trypsinogen (*PRSS3*) with vector, EK^*WT*^, EK^*Val*799*Asp*^ to allow EK to activate trypsinogen. Then, the activity of trypsin was detected. The results showed the activities of EK^*Val*799*Asp*^ decreased by 37%, compared with that of EK^*WT*^ (Figure 4B).

## Discussion

EKD is an extremely rare inherited disorder. All the reported patients presented with diarrhea, hypoproteinemia and no increase in weight (Haworth et al., 1971; Hadorn et al., 1975; Ghishan et al., 1983; Green et al., 1984), and 50% of the affected children had vomiting, edema and anemia. Biopsy of the proximal duodenum was histologically normal. The patients usually had clinical manifestations after birth; however, Haworth et al. (1971), Holzinger et al. (2002) reported that one patient was asymptomatic before the age of 5 months, and his first symptoms appeared after weaning and feeding with cow’s milk and cereal. The patient described in the present study was similar to this child, based on clinical presentation and age of onset.

In 2002, Holzinger et al. (2002) sequenced two families with EKD reported by Haworth et al. (1971) in 1971 and Hadorn et al. (1975) in 1975, and they found that the compound heterozygous variants in the *TMPRSS15* gene were responsible for the onset of EKD. The *TMPRSS15* gene, formerly known as *PRSS7* or *ENTK*, is located on chromosome 21q21.1, with a total length of 134.54 kb, containing 25 exons and encoding 1019 amino acids (Kitamoto et al., 1994, 1995). This gene encodes enterokinase (synonym: enteropeptidase), a type II transmembrane serine protease, which is expressed only at the brush border of the proximal small intestine. Human EK is composed of a transmembrane domain, a heavy chain and a light chain. The light chain is a serine protease domain with histidine, aspartic acid and serine active sites. The heavy chain is composed of repeated motifs that are homologous to domains of other proteins (Figure 2A; Zheng et al., 2009). EK activates trypsinogen by cutting the inhibitory part to initiate the protease activation cascade of digestive enzymes and catalyze the conversion of inactive trypsinogen to active trypsin (Yamashina, 1956). Trypsin further activates chymotrypsin, elastase, carboxypeptidase A and B, lipase, etc., allowing proteins to be absorbed (Kitamoto et al., 1994).

Due to the rarity of the disease and the limitations of diagnostic methods, only four genetic variations in the *TMPRSS15* gene have been reported in the HGMD so far. The compound heterozygous nonsense variants c. 2135C > G;p.(Ser712^∗^) and c.2569C > T;p.(Arg857^∗^) in two siblings of a Western European family led to premature termination of the codon at Ser712 in the macrophage scavenger receptor-like domain (MSCR) domain and Arg857 in the serine protease domain on the heavy chain (Holzinger et al., 2002; Zheng et al., 2009). Another reported patient had the heterozygous nonsense variant c.781C > T [p.(Gln261^∗^)] in exon 8 and the heterozygous deletion variant c.2707_2708del [p.(Val903Phefs^∗^29)] in exon 23, which resulted in premature termination of Gln261 in the heavy C1r/s motif and frameshift of amino acids in the serine protease domain, respectively (Kitamoto et al., 1994; Holzinger et al., 2002). ClinVar included two additional variants (c.151_155del and c.1428 + 2T > G), but no clinical data were available.

We reported a Chinese patient with two novel *TMPRSS15* variants in compound heterozygosity: a splicing change c.1921G > A causing exon 16 skipping, and p.Val799Asp missense change reducing both expression and enzymatic activity. The skipping of exon 16 would generate an alternative transcript without exon 16 (*TMPRSS15*-d16), resulting in an in-frame 47-amino-acid deletion (p.Ala594_Pro640del). This protein may maintain a partial enzymatic function, explaining the finding of three homozygotes for the c.1921G > A variant in GnomAD database. It could also be possible that the c.1921G > A variant is leading to an incomplete exon skipping *in vivo* and the formation of a partially functional EK protein with the p.Glu641Lys change. Furthermore, we suggest that some cases may not manifest a severe phenotype in childhood. And Ghishan et al. (1983) reported that patients could have no relevant clinical manifestations after complete remission.

Though the molecular basis of the compound heterozygous variants in *TMPRSS15* in this patient was elucidated by *in vitro* functional studies, there are still some clinical questions that need to be addressed. Why did the patient develop such severe phenotype? Why was vomiting significantly alleviated under fasting while diarrhea was not? Combined with the stool analysis that showed an increase in leucocytes and erythrocytes, we think the child also had an intestinal infection. Infection and withdrawal of breast milk affected the compensatory function of the residual enzyme activity. Meanwhile, complications of intestinal and pulmonary infections worsen EKD. Initially, we failed to identify the cause of diarrhea, and the patient did not receive timely trypsin treatment, resulting in prolonged diarrhea and eventually intestinal villus injury and intestinal flora disorder. These factors are responsible for the severe phenotype.

Although EKD is a rare congenital disease, the reported patients responded well to pancreatic enzyme replacement therapy. However, some patients experience diarrhea and other symptoms in adolescence after complete remission and return to normal after receiving the dose of pancreatic enzyme replacement therapy is increased (Hadorn et al., 1975). An explanation for this self-healing tendency and recurrence might be the decrease in individual demand for protein digestion with increasing age. The feedback mediated by trypsin or other activation pathways independent of trypsin and EK is sufficient to meet adult protein digestion requirements (Holzinger et al., 2002). However, it is not enough for protein digestion in infants, and recurrence may be associated with increased protein demand during the growth spurt of adolescence (Holzinger et al., 2002).

Carrier parents can be informed about the 25% risk of EKD in future pregnancies. Prenatal screening can be offered, and couples should be aware that EKD is a disease with a good prognosis. However, in case of birth of an EKD patient, parents should pay attention to the feeding problem and provide pancreatic exocrine secretion treatment appropriately. The typical diagnosis of EKD was based on the detection of trypsin activity in duodenal fluid. Because of the difficulty in obtaining the contents of the duodenal intestine and measuring EK activity, patients could be misdiagnosed, or the right treatment could be delayed. Currently, with the application of high-throughput sequencing and the understanding of the genetic mechanism of the disease, genetic analysis provides an efficient method for early diagnosis of EKD. When infants with chronic diarrhea, hypoproteinemia and normal intestinal histopathology fail to respond to conventional treatment, we should consider EKD in differential diagnosis. These children can be treated with pancreatic exocrine secretion even before genetic reports are available. Early diagnosis and intervention can improve the survival rate and ensure a better quality of life for patients suffering from this inherited disease.

## Data Availability Statement

All datasets generated for this study are included in the article/Supplementary Material, further inquiries can be directed to the corresponding authors.

## Ethics Statement

The studies involving human participants were reviewed and approved by the ethics committee of the Children’s Hospital of Nanjing Medical University. Written informed consent to participate in this study was provided by the participants’ legal guardian/next of kin. Written informed consent was obtained from the individual(s), and minor(s)’ legal guardian/next of kin, for the publication of any potentially identifiable images or data included in this article.

## Author Contributions

ZL, BZ, LZ, and YJ conceived and designed this study. LW and DZ wrote the manuscript. LW and XZ performed the experiments. DZ and CF collected the clinical samples and clinical data. BZ performed NGS analysis. ZL, LZ, and YJ reviewed and edited the manuscript.

## Conflict of Interest

The authors declare that the research was conducted in the absence of any commercial or financial relationships that could be construed as a potential conflict of interest.

## Footnotes

1 http://genetics.bwh.harvard.edu/pph2/

2 https://sift.bii.a-star.edu.sg/

3 http://fathmm.biocompute.org.uk/inherited.html

4 http://consurf.tau.ac.il/

5 http://www.umd.be/HSF/

6 http://www.cbs.dtu.dk/services/NetGene2

7 http://www.fruitfly.org/seq_tools/splice.html

8 https://www.ncbi.nlm.nih.gov/tools/primer-blast/index

## Supplementary Material

FIGURE S1 The timeline of clinical phenotype development. The black arrow in the center represents the timeline. The clinical manifestations, examinations, and therapies at corresponding time are shown from top to bottom. WBC, white blood cell; WES, whole exome sequencing; Stool-RT, stool routine; eHF, extensively hydrolyzed formula.

FIGURE S2 The minigene splicing assay of the c.1921G > A variant *in vitro* and the transfection efficiency of overexpressed plasmids. **(A)** Gel electrophoresis of the RT-PCR product of minigene transcripts in A549 and HeLa cells. Lane 1 and 5: DL1000 Plus DNA Marker (Vazyme, China); lane 2 and 6: pSPL3 (263 bp); lane 3 and 7: E16-WT (404 bp); lane 4 and 8: c.1921G > A (263 bp). **(B)** The relative mRNA expression of pcDNA3.1-3xFlag-*TMPRSS15*, pcDNA3.1-3xFlag-*TMPRSS15*-MUT in HEK293 cells were assessed by RT-qPCR using *GAPDH* gene as reference gene. The experiments were repeated at least three times.
