# Supplementary figures and images for: Novel Compound Heterozygous TMPRSS15 Gene Variants Cause Enterokinase Deficiency
Source: Front Genet. 2020 Sep 11;11:538778. doi: 10.3389/fgene.2020.538778 (PMC7517701; doi:10.3389/fgene.2020.538778)

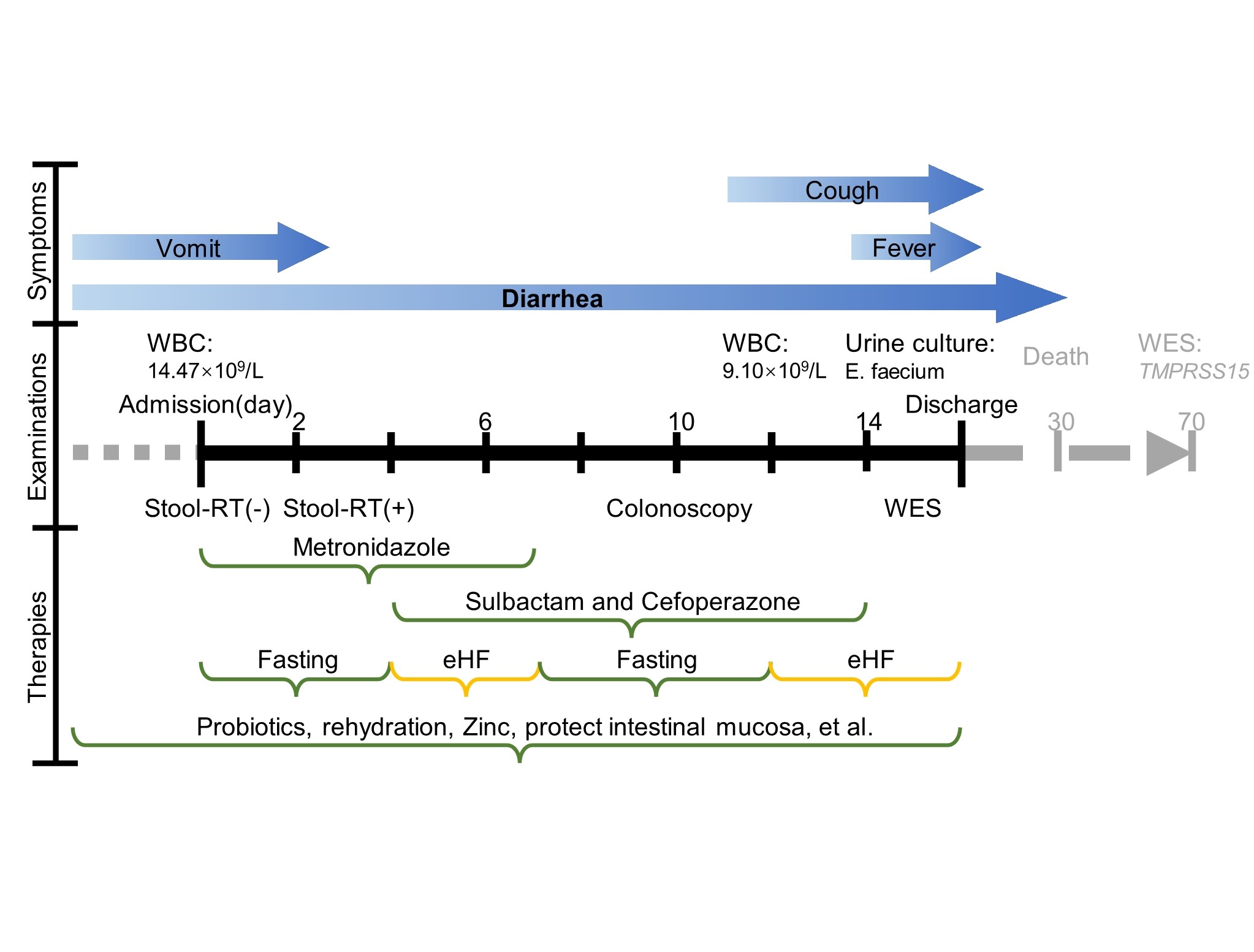

Supplement: FIGURE S1 — The timeline of clinical phenotype development. The black arrow in the center represents the timeline. The clinical manifestations, examinations, and therapies at corresponding time are shown from top to bottom. WBC, white blood cell; WES, whole exome sequencing; Stool-RT, stool routine; eHF, extensively hydrolyzed formula. [file Image_1.jpg]

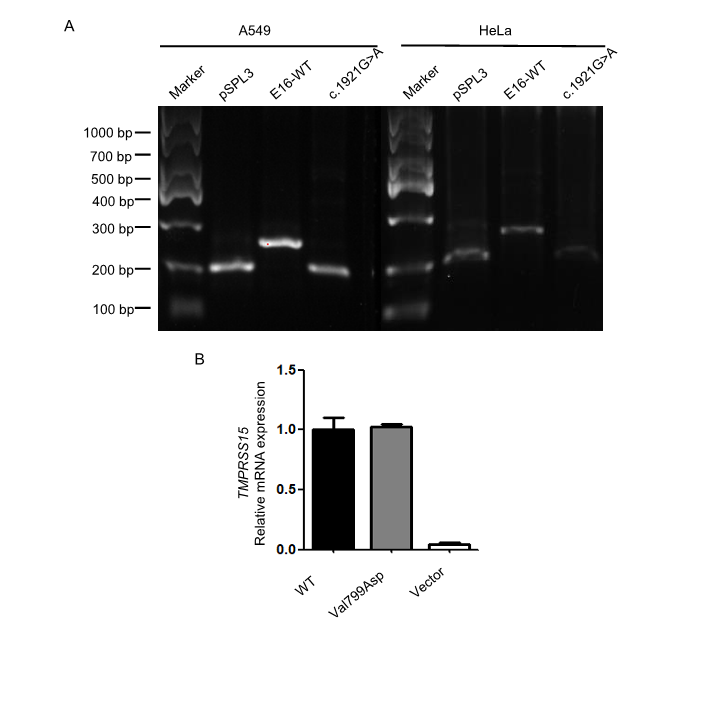

Supplement: FIGURE S2 — The minigene splicing assay of the c.1921G > A variant in vitro and the transfection efficiency of overexpressed plasmids. (A) Gel electrophoresis of the RT-PCR product of minigene transcripts in A549 and HeLa cells. Lane 1 and 5: DL1000 Plus DNA Marker (Vazyme, China); lane 2 and 6: pSPL3 (263 bp); lane 3 and 7: E16-WT (404 bp); lane 4 and 8: c.1921G > A (263 bp). (B) The relative mRNA expression of pcDNA3.1-3xFlag-TMPRSS15, pcDNA3.1-3xFlag-TMPRSS15-MUT in HEK293 cells were assessed by RT-qPCR using GAPDH gene as reference gene. The experiments were repeated at least three times. [file Image_2.TIF]
